# Subtypes, resistance and virulence platforms in extended-drug resistant *Acinetobacter baumannii* Romanian isolates

**DOI:** 10.1038/s41598-021-92590-5

**Published:** 2021-06-24

**Authors:** Irina Gheorghe, Ilda Czobor Barbu, Marius Surleac, Ionela Sârbu, Laura Ioana Popa, Simona Paraschiv, Yu Feng, Veronica Lazăr, Mariana Carmen Chifiriuc, Dan Oţelea, Zong Zhiyong

**Affiliations:** 1 Department of Microbiology and Immunology, Faculty of Biology, University of Bucharest, Bucharest, Romania; 2 Research Institute of the University of Bucharest (ICUB), Bucharest, Romania; 3 Genetics Department, Faculty of Biology, University of Bucharest, Bucharest, Romania; 4 Department of Bioinformatics, National Institute of Research and Development for Biological Sciences, Bucharest, Romania; 5 National Institute for Infectious Diseases “Matei Bals”, Bucharest, Romania; 6 Centre of Infectious Diseases, West China Hospital, Sichuan University, Chengdu, China; 7 Academy of Romanian Scientists, 050045 Bucharest, Romania

**Keywords:** Microbiology, Infectious diseases

## Abstract

*Acinetobacter baumannii* has emerged worldwide as a dominant pathogen in a broad range of severe infections, raising an acute need for efficient antibacterials. This is the first report on the resistome and virulome of 33 extended drug-resistant and carbapenem-resistant *A. baumannii* (XDR CRAB) strains isolated from hospitalized and ambulatory patients in Bucharest, Romania. A total of 33 isolates were collected and analyzed using phenotypic antibiotic susceptibility and conjugation assays, PCR, whole-genome sequencing (WGS), pulsed-field gel electrophoresis (PFGE) and MultiLocus Sequence Typing (MLST). All isolates were extensively drug-resistant (XDR), being susceptible only to colistin. The carbapenem resistance was attributed by PCR mainly to *bla*_OXA-24_ and *bla*_OXA-23_ genes. PFGE followed by MLST analysis demonstrated the presence of nine pulsotypes and six sequence types. WGS of seven XDR CRAB isolates from healthcare-associated infections demonstrated the high diversity of resistance genes repertoire, as well as of mobile genetic elements, carrying ARGs for aminoglycosides, sulphonamides and macrolides. Our data will facilitate the understanding of resistance, virulence and transmission features of XDR AB isolates from Romanian patients and might be able to contribute to the implementation of appropriate infection control measures and to develop new molecules with innovative mechanisms of action, able to fight effectively against these bugs, for limiting the spread and decreasing the infection rate and mortality.

## Introduction

*Acinetobacter baumannii* is one of the most frightening resistant Gram-negative bacteria, being included in both the ESKAPE (later ESCAPE) group and the list of 12 bacteria that represent a threat to human health published in 2017 by the World Health Organization (WHO). The successful development of efficient antibacterials requires a better knowledge of the mechanisms of resistance of this pathogen, to increase the chance to discover new chemical structures with ideally new mechanisms of action^1^ or different antimicrobial combinations^2, 3^.

Several mechanisms contribute to *A. baumannii* strains resistance, such as β-lactamases expression, alteration of cell membrane permeability, increased expression of efflux pumps, mutations in DNA gyrases and topoisomerases encoding genes^3–10^. The most frequent causes of enzymatic resistance in *A. baumannii* are represented nowadays by the acquired Carbapenem-Hydrolyzing Class D β-Lactamases (CHDL) (OXA-23, OXA-24, OXA-58, OXA-143, OXA-235) and the overexpression of the intrinsic OXA-51, followed by the presence of class A β-lactamases (i.e. TEM (for Temoneira patient’s name), SHV (Sulfhydryl Variable enzyme), CTX-M (Cefotaxime first isolated in Munich) and KPC (*Klebsiella pneumoniae* Carbapenemase*)*, GES (Guyana Extended Spectrum β-lactamase), class A extended-spectrum cephalosporinases (usually caused by the over-production of the chromosomal ADCs) and metallo-β-lactamases (MBL) (IMP (active-on-imipenem), VIM (Verona imipenemase), SIM (Seoul imipenemase), SPM (Saõ Paolo metallo-β-lactamase), GIM (German imipenemase) and NDM (New Delhi MBL)^11^. The expression of OXA genes might be increased by insertion sequences (ISs), such as IS*Aba1*, IS*Aba4* and IS*Aba125*, which provide an additional strong promoter^12, 13^. The insertion sequence IS*Aba1* has been found in *A. baumannii* isolates upstream of the *bla*_OXA-23,-51,-58_ carbapenemase and cephalosporinase *bla*_ampC_ genes^14^. Among different genetic contexts containing OXA-23 and involving transposons, such as Tn*2006*, Tn*2008*, Tn*2008B14*, Tn*2009* and Tn*2007*, only Tn*2006* and Tn*2009* are compound transposons, containing two copies of IS*Aba1* flanking the carbapenemase gene and thus able to regulate its mobility. In the case of the Tn*2008* and Tn*2008B*, there is only one copy of the IS*Aba1* upstream *bla*_OXA-23_ gene^12, 15–17^.

Carbapenem-resistant *A. baumannii* (CRAB) is a globally disseminated pathogen with continuously increasing prevalence worldwide. CRAB strains are frequently exhibiting MDR and XDR resistance phenotypes, carrying both intrinsic and acquired resistance genes carried on plasmids, transposons, and integrons^18^. In Romania, the present knowledge on the distribution and molecular epidemiology of CRAB isolates is scarce and limited to the study of low numbers of clinical strains recovered from the north, west, and south regions of the country. In this context, we aim to investigate the resistance and virulence features of 30 clinical strains and 3 community-acquired XDR CRAB, by phenotypic antibiotic susceptibility assays, PCR, pulsed-field gel electrophoresis (PFGE), MultiLocus Sequence Typing (MLST) and whole-genome sequencing (WGS).

## Results

### Antimicrobial resistance profiles of CRAB

The great majority of the *A. baumannii* isolates (96.96%) were resistant to imipenem, meropenem (MIC established by broth microdilution method of 8 mg/L) and ciprofloxacin. High resistance rates were recorded for cefepime (93.93%), trimethoprim-sulfamethoxazole, gentamicin and cefoxitin (90.90% each) and levofloxacin (87.87%). Lower rates were registered for piperacillin-tazobactam (42.42%) and ceftriaxone (39%) (Table 1).

**Table 1:** Clinical specimen, carbapenemase, PFGE-type and MLST results, for *A. baumannii* clinical isolates from Romania.

Isolate	Source	Clinical Unit/Date	PFGE	MLST	Acquired carbapenemase	Antibiotic Resistance profile
*A17*	*Blood culture*	*A, January 2018*	*III*	ST2	OXA-23*, OXA-51	MEM, IMP, ETP, CEF, CXM, CTX, CAZ, CIP, FOX, FEP, ATM, AMC, PIP-TZP, SXT, AMK, GEN, NIT, LEV, TIG
*A15*	*Pharyngeal exudate*	*A, January 2018*	*II*	ST2	OXA-23*, OXA-51	MEM, IMP, ETP, CEF, CXM, CTX, CAZ, CIP, FOX, FEP, ATM, AMC, PIP-TZP, SXT, AMK, GEN, NIT, LEV, TIG
*4542*	*Pharyngeal exudate*	*A, December 2017*	*I*	ST2	OXA-23*, OXA-51	MEM, IMP, ETP, CEF, CXM, CTX, CAZ, CIP, FOX, FEP, ATM, AMC, PIP-TZP, SXT, AMK, GEN, NIT, LEV, TIG
*A9*	*Stool culture*	*A, January 2018*	*II*	ST2	OXA-23*, OXA-51	MEM, IMP, ETP, CEF, CXM, CTX, CAZ, CIP, FOX, FEP, ATM, AMC, PIP-TZP, SXT, AMK, GEN, NIT, LEV, TIG
*A11*	*Anal carriage*	*A, February 2018*	*I*	ST2	OXA23*, OXA-51	MEM, IMP, ETP, CEF, CXM, CTX, CAZ, CIP, FOX, FEP, ATM, AMC, PIP-TZP, SXT, AMK, GEN, NIT, LEV, TIG
*A07*	*Blood culture*	*A, December 2017*	*II*	ST2	OXA-23*, OXA-51	MEM, IMP, ETP, CEF, CXM, CTX, CAZ, CIP, FOX, FEP, ATM, AMC, PIP-TZP, SXT, AMK, GEN, NIT, LEV, TIG
*A6*	*Stool culture*	*A, November 2017*	*II*	ST2	OXA-23*, OXA-51	MEM, IMP, ETP, CEF, CXM, CTX, CAZ, CIP, FOX, FEP, ATM, AMC, PIP-TZP, SXT, AMK, GEN, NIT, LEV, TIG
*A12*	*Anal carriage*	*A, February 2018*	*﻿I*	ST2	OXA-51, OXA-23	MEM, IMP, ETP, CEF, CXM, CTX, CAZ, CIP, FOX, FEP, ATM, AMC, PIP-TZP, SXT, AMK, GEN, NIT, LEV, TIG
*A14*	*Blood culture*	*A, February 2018*	*I*	ST2	OXA-23*, OXA-51	MEM, IMP, ETP, CEF, CXM, CTX, CAZ, CIP, FOX, FEP, ATM, AMC, PIP-TZP, SXT, AMK, GEN, NIT, LEV, TIG
*A10*	*Stool culture*	*A, December 2017*	*II*	ST2	OXA-23*, OXA-51	MEM, IMP, ETP, CEF, CXM, CTX, CAZ, CIP, FOX, FEP, ATM, AMC, PIP-TZP, SXT, AMK, GEN, NIT, LEV, TIG
*A13*	*Anal carriage*	*A, February 2018*	*V*	ST642	OXA-24, OXA-51	MEM, IMP, ETP, CEF, CXM, CTX, CAZ, CIP, FOX, FEP, ATM, AMC, PIP-TZP, SXT, AMK, GEN, NIT, LEV, TIG
*A1*	*Blood culture*	*A, October 2017*	*IV*	ST312	OXA-24, OXA-51	IPM, MEM, AMC, CTX, CAZ, PIP-TZP, CIP, LEV, GEN, AMK
*A1prim*	*Blood culture*	*A, October 2017*	*IV*	ST312	OXA-24, OXA-51	IPM, MEM, AMC, CTX, CAZ, PIP-TZP, CIP, LEV, GEN, AMK
*8A*	*Urine*	*B, September 2017*	*II*	ST2	OXA-23*, OXA-51	IPM, MEM, PIP-TZP, CAZ, FEP, ATM, AMK, GEN, CIP
*7A*	*Urine*	*B, September 2017*	*IX*	ST2	OXA-24, OXA-51	IPM, CAZ, FEP, FOX, AMK, GEN, CIP, LEV, SXT, TET
*6A*	*bBood culture*	*B, September 2017*	*VIII*	ST636	OXA-24, OXA-51	IPM, MEM, CAZ, FEP, FOX, AMK, GEN, CIP, LEV, SXT, TET
*22s*	*Tracheal secretion*	*C, September 2017*	*VI*	ST636	OXA-24, OXA-51	AMK, CAZ, FEP, CIP, PIP, IPM, MEM, SXT, FOX, LEV
*24s*	*Tracheal secretion*	*C, September 2017*	*VI*	ST636	OXA-24, OXA-51	AMK, CAZ, FEP, CIP, GEN, PIP, IPM, MEM, SXT, FOX, LEV, TET
*21s*	*Catheter*	*C, September 2017*	*VI*	ST636	OXA-24, OXA-51	AMK, CAZ, FEP, CIP, GEN, PIP, IPM, MEM, SXT, FOX,
*18s*	*Catheter*	*C, September 2017*	*VI*	ST1	OXA-24*, OXA-51	AMK, CAZ, FEP, CIP, GEN, PIP, IPM, MEM, SXT, FOX, LEV, TET
*19s*	*Urine*	*C, September 2017*	*VII*	ST492	OXA-24, OXA-51*	AMK, CAZ, FEP, CIP, GEN, PIP, IPM, MEM, SXT, FOX, LEV, TET
*29s*	*Tacheal secretion*	*C, September 2017*	*VII*	ST492	OXA-24, OXA-51	AMK, CAZ, FEP, CIP, GEN, PIP, IPM, MEM, SXT, FOX, LEV, TET
*2s*	*Tracheal secretion*	*C, September 2017*	*VI*	ST636	OXA-24, OXA-51	AMK, CAZ, FEP, CIP, GEN, PIP, IPM, MEM, SXT, FOX, LEV, TET
*33s*	*Tracheal secretion*	*C, September 2017*	*VII*	ST492	OXA-24, OXA-51	AMK, CAZ, FEP, CIP, GEN, PIP, IPM, MEM, SXT, FOX, LEV, TET
*9s*	*Tracheal secretion*	*C, September 2017*	*VII*	ST492	OXA-24, OXA-51*	AMK, CAZ, FEP, CIP, GEN, PIP, IPM, MEM, SXT, FOX, TET
*14s*	*Catheter*	*C, September 2017*	*VII*	ST492	OXA-24, OXA-51*	AMK, CAZ, FEP, CIP, GEN, PIP, IPM, MEM, SXT, FOX, LEV, TET
*01s*	*Cerebrospinal fluid*	*C, September 2017*	*VI*	ST636	OXA-24, OXA-51	AMK, CAZ, FEP, CIP, GEN, PIP, IPM, MEM, SXT, FOX, LEV, TET
*3s*	*Catheter*	*C, September 2017*	*VI*	ST636	OXA-24, OXA-51	AMK, CAZ, FEP, CIP, PIP, IPM, MEM, SXT, FOX, LEV, TET
*4s*	*Tracheal secretion*	*C*	*negative*		OXA-24, OXA-51	AMK, CAZ, FEP, CIP, PIP, IPM, MEM, SXT, FOX, LEV, TET
*10s*	*Tracheal secretion*	*C, September 2017*	*negative*	ST492	OXA-24*, OXA-51	AMK, CAZ, FEP, CIP, GEN, PIP, IPM, MEM, SXT, FOX, TET
*26s*	*Tracheal secretion*	*C, September 2017*	*IV*	ST312	OXA-24, OXA-51	AMK, CAZ, FEP, CIP, GEN, PIP, IPM, MEM, SXT, FOX, LEV, TET
*24s prim*	*Tracheal secretion*	*C, September 2017*	*VI*	ST636	OXA-24, OXA-51	AMK, CAZ, FEP, CIP, GEN, PIP, IPM, MEM, SXT, FOX, LEV, TET
*4 new*	*Sputum*	*B, February 2018*	*VI*	ST636	OXA-24, OXA-51	MEM, FEP, GEN, SXT, LEV, TET

^1^ Notes: A = ICU ; B- Ambulatory; C- Children Hospital. (*) indicates the IS*Aba1* insertion upstream of the gene encoding respective carbapenemase.

MEM = meropenem, IPM = imipenem, ETP = ertapenem, CEF = cephalotin, CTX = ceftriaxone, CXM = cefuroxime, FOX = cefoxitin, CAZ = ceftazidime, ATM = aztreonam, FEP = cefepime, AMC = amoxicillin-clavulanic acid, PIP-TZP = piperacillin-tazobactam, CIP = ciprofloxacin, LEV = levofloxacin, GEN = gentamicin, AMK = amikacin, NIT = nitrofurantoin, SXT = trimethoprim-sulfamethoxazole, TET = tetracycline, TIG = tigecycline.

### Detection of CHLDs genes

The *A. baumannii* isolates were investigated for the presence of OXA-type carbapenemases (Table 1). All isolates harbored the intrinsic *bla*_OXA-51-like_ gene, 66.66% of *A. baumannii* revealed *bla*_OXA-24_ and 33.33% of *A. baumannii* isolates carried *bla*_OXA-23_. We did not detect any MBL gene. The presence of IS*Aba1* was confirmed in 15 isolates (45.45%) and 10 strains (30.30%) had IS*Aba1* immediately upstream of the *bla*_OXA-23_ gene. No IS*Aba1* was detected downstream of the *bla*_OXA-23_ gene, ruling out the hypothesis that this gene could be part of the composite transposon Tn*2006*.

### Epidemiological typing of isolates by PFGE and MLST

Nine major genotypes were encountered in the analyzed *A. baumannii* strains using a cut-off of 85% genetic similarity (Table 1): pulsotype I (n = 4), II (n = 6), III (n = 1), IV (n = 3), V (n = 1), VI (n = 9), VII (n = 5), VIII (n = 1) and IX (n = 1), while 2 strains were non-typeable. No correlation could be established between the origin of the analyzed strains (a certain clinical unit) and a specific pulsotype. The MLST analysis performed for the selected clones showed that only 12 of the 33 isolates belonged to the most prevalent global Clonal Complex CC2 (ST2) and 5 singleton STs were identified (ST636, ST492, ST312, ST642, and ST1) (Table 1). The PFGE results have shown that the most common clones were VI (n = 9), II (n = 6), VII (n = 5), I (n = 4) (Table 1).

The PFGE results revealed that there was no outbreak or spread of one single genotype in the clinical strains that were analyzed. However, clones I, II, III, IV and V were associated with hospital A, clones II, VIII and IX with the ambulatory unit B and clones IV, VI and VII were found only in hospital C.

### Genomic analyses

The genomes of 7 selected CRAB strains encoded 01s, 14s, 10s, 18s, 24s, A07 and A14 were fully sequenced and analyzed to have a complete picture of the antibiotic resistance and virulence genes repertoires. The analyzed strains were selected for WGS based on the isolation source, all being isolated from healthcare-associated infections, respectively catheter-associated bloodstream infections, ventilator-associated pneumonia and central nervous system infections. Draft-genome sequencing analysis revealed that the chromosome size varied, as expected, between 3.86 and 4.071 Mbp. Instead, the analyzed strains harbored diverse mobile genetic elements. More than half of the CDS were functionally annotated by the RAST program. The general features of the genomes are presented in Table 2.

**Table 2:** General features of *A. baumannii* strains genomes.

Strain	01s	14s	10s	18s	24s	A07	A14
Size	3,968,270	3,897,785	3,860,242	3,977,351	3,925,255	3,970,732	4,071,708
GC Content (%)	39.04	39.03	38.98	39.14	39.0	39.07	38.88
Contig N50	161,906	162,094	132,810	81,560	107,477	127,142	135,471
Contig L50	9	8	10	16	12	10	10
Number of Contigs (with PEGs)	166	128	130	232	181	158	205
Number of Coding Sequences	3908	3784	3770	3931	3836	3907	4061
Genes assigned to COs	2173	2148				2165	2158
Number of RNAs	71	65	87	70	70	78	69

To analyze the acquired antibiotic resistance genes (ARGs), ResFinder version 3.0 with an ID threshold of 90%, and the minimum length set at 60% were used. The analysis revealed that all strains harbor acquired ARGs to aminoglycosides (i.e. *aph(3')-*VIa, *ant(3'')-IIa*, *aph(6)-Id*, *aph(3'')-Ib*, *aadA1*, *aadA2*, *armA*, *aph(3')VIj*, *ant(3'')-IIa*, *aph(3')-Ia*, *aadA1*, *aac(3)-Ia)*, β-lactams (*bla*_OXA-66_, *bla*_OXA-72_, *bla*_OXA-92_, *bla*_OXA-23_, *bla*_ADC-25_, *bla*_ADC-30_, *bla*_ADC-81_, *bla*_ADC-74_, *blaTEM-1D*) and sulphonamides (*sul1*/ *sul2* or both of them), while 4 strains carry ARGs for tetracyclines [*tet*(*B*) and *tetR* encountered in 3 strains from hospital settings A and C and *tet*(*A*) gene revealed in one strain]. The predictions were carried with multiple tools as described above, to increase the confidence degree in the predicted genes. The *catA1* gene associated with resistance to chloramphenicol was located between Tn*As3* and IS*1R* in 2 strains. ARGs for streptogramin B [*msr*(*E*)], *mph*(*E*)] have been revealed also in 2 strains from the hospital settings A and C (Table 3).

**Table 3:** AR and virulence genetic markers in the analyzed *A. baumannii* strains.

		01s	14s	10s	18s	24s	A07	A14
Antibiotic resistance	Aminoglycosides	*aph(3')-Ia*, *aph(3')-VIa*, *ant(3'')-IIa*	*aph(6)-Id*, *ant(3'')-IIa*, *aph(3'')-Ib*, *aadA2*, *armA*	*aph(3'')-Ib*, *aph(6)-Id*, *ant(3'')-IIa*,	*aph(3')-VIa*, *ant(3'')-IIa*, *aac(6')-Ib*, *aac(3)-I*, *aadA1*, *ant(3'')-IIa*	*aadA1*, *aph(3')-Ia*, *aph(3')-VIa*, *ant(3'')-IIa*,	*aph(3')VIb*, *ant(3'')-IIa*, *aph(3')-Ia*, *aadA1*, *aac(3)-Ia*	*aph(6)-Id*, *ant(3'')-IIa*, *aph(3'')-Ib*, *armA*
	β-lactams	*bla*_OXA-66_, *bla*_OXA-72_, *bla*_ADC-74_	*bla*_OXA-66_, *bla*_OXA-72_, *bla*_ADC-25_	*bla*_OXA-66_, *bla*_OXA-72_, *bla*_ADC-30_	*bla*_OXA-92_, *bla*_ADC-81_, *bla*_OXA-72_, *bla*_TEM-84_, *bla*_TEM-12_,	*bla*_OXA-72_, *bla*_OXA-66_, *bla*_ADC-74_,	*bla*_OXA-66_, *bla*_ADC-11_, *bla*_TEM-12_, *bla*_PER-1_	*bla*_OXA-66_, *bla*_OXA-23_, *bla*_ADC-11_, *bla*_PER-1_
	Macrolides		*msr*(*E*), *mph*(*E*)					*msr*(*E*), *mph*(*E*)
	Tetracycline		*tet*(*B*), *tetR*	*tet*(*B*),	*tet*(*A*)			*tet*(*B*),
	Phenicol	*catA1*			*catA1*	*catI*		
	Folate inhibitors	*sul1*	*dfrA12*, *sul1*, *sul2*	*sul2*	*sul1*	*sul1*	*sul1*	*sul1*
	Quinolones resistance	*gyrA* (S83L), *parC* (S84L)	*gyrA* (S83L), *parC* (S84L), *parC* (S467G)	*gyrA* (S83L), *parC* (S84L), *parC* (S467G)	*gyrA* (S83L), *parC* (S84L), *parC* (S467G)	*gyrA* (S83L), *parC* (S84L)	*gyrA* (S83L, *parC* (S84L), *parC* (S467G)	*gyrA* (S83L), *parC* (S84L), *parC* (S467G)
	antibiotic efflux	*abeM*, -*S*; *adeABC*, -*FGH*, -*IJK*, -*L*, -*N*, -*R*, -*S*	*abeM*, -*S*; *adeABC*, -*FGH*, *-IJK*, -*L*, -*N*, -*R*, -*S*	*abaF*, -*Q*; *adeK*, -*I*, -*S*, -*L*, -*G*, -*A*, -*C*, -*N*, -*F*, -*H*, *-R*, *amvA*	*abaQ*; *adeF*, -*S*, -*G*, -*I*, -*K*, -*N*, -*H*, -*L*, *amvA*	*adeA*, -*C*, -*S*, -*L*, -*G*, -*K*, -*I*, -*R*, -*F*, -*H*, -*N*; amvA; AbaF, -Q,	*abeM*, -*S*; *adeABC*, -*FGH*, -*IJK*, -*L*, -*N*, -*R*, -*S*	*abeM*, -*S*; *adeABC*, -FGH, -*IJK*, -*L*, -*N*, -*R*, -*S*
Virulence factors	Adherence	*ompA*	*ompA*	*ompA*	*ompA*	*ompA*	*ompA*	*ompA*
	Biofilm formation	*adeFGH*, *bap*, *csuA*/*BABCDE*, *pgaABCD*,	*adeFGH*, *bap*, *csuA*/*BABCDE*, *pgaABCD*	*adeFGH*, *bap*, *csuA*/*BABCDE*, *pgaABCD*	*adeFGH*, *bap*, *csuD*, *csuE*, *pgaABCD*	*adeFGH*, *bap*, *csuA*/*BABCDE*, *pgaABCD*	*adeFGH*, *bap*, *csuA*/*BABCDE*, *pgaABCD*	*adeFGH*, *bap*, *csuA*/*BABCDE*, *pgaABCD*
	Enzyme	*plc*, *plcD*	*plc*, *plcD*	*plc*, *plcD*	*plc*, *plcD*	*plc*, *plcC*, *plcD*	*plc*, *plcD*	*plc*, *plcD*
	Pore forming toxins	*apkA*, *apkB*	*apkA*, *apkB*	*apkA*, *apkB*	*apkA*, *apkB*	*apkA*, *apkB*	*apkA*, *apkB*	*apkA*, *apkB*
	Iron uptake	*tonB*	*tonB*, *hemO*	*tonB*	*tonB *	*tonB*	*tonB*	*tonB*, *hemO*
Resistance to toxic compounds	Copper	*MmcO*, CtpA*, Bcr/CflA, cueR, pcoB, cutE, CorC*	*MmcO*,* CtpA, Bcr/CflA, cueR, pcoB, cutE, CorC*	*MmcO*,* CtpA,, Bcr/CflA, cueR,* *pcoB, cutE, CorC*	*MmcO*,* CtpA, Bcr/CflA, cueR,* *pcoB, pcoD,* CusR,* CopC, cutE, CorC*	*MmcO*,* CtpA,, Bcr/CflA, cueR,* * pcoB, cutE, CorC*	*MmcO*,* CtpA, Bcr/CflA, cueR, pcoB, cutE, CorC*	*MmcO*,* CtpA, Bcr/CflA, cueR, pcoB, cutE, CorC*
	Cobalt, Zinc, Cadmium	*CzcABCD*, *MFP*, *merR*, *CusA*	*CzcABD*, *MFP*, *merR*, *CusA*	*CzcABD*, *MFP*, *merR*, *CusA*	*CzcABCD*, *MFP*, *merR*, *CusA*, *CusR*, *CusS*	*CzcABCD*, *MFP*, *merR*, *CusA*	*MFP*, *czcABD*, *merR*, *CusA*	*MFP*, *czcABD*, *merR*, *CusA*
	Chromium	*ChrA*	*ChrA*	*ChrA*	*ChrA*	*ChrA*	*ChrA*	*ChrA*
	Mercury				*merA*, *merC*, *merR*			
	Arsenic	*ACR3*, *arsC*, *arsR*	*ACR3*, *arsC*, *arsR*	*ACR3*, *arsC*, *arsR*	*ACR3*, *arsC*, *arsH*, *arsR*	*ACR3*, *arsC*, *arsR*	*ACR3*, *arsC*, *arsH*, *arsR*	*ACR3*, *arsC*, *arsR*
Integron		Incomplete (3’-CS region only)	5’-CS – *dfrA12 – aadA2 – *3’-CS	–	5’-CS – *aac(3)-I – aadA1 – *3’CS	5’-CS – *aac(3)-I – aadA1 – *3’CS	5’-CS – *ac(3)-I – aadA1 – *3’CS	–
Pathogen Finder		0.853	0.852	0.852	0.845	0.852	0.852	0.854
Plasmid replicons and carried antibiotic resistance genes		GR6 (pACICU2-like – AN: CP031382.1) - *aph(3')-VIa*	pMAL-1 like (AN: NZ_KX230793.1) - *bla*_OXA-72_,	pMAL-1 like - *bla*_OXA-72_	pMAL-1 like - *bla*_OXA-72_ pESBL234 like (AN: NZ_MT230226.1) – *tet(A)*	GR6 (pACICU2-like: ) - *aph(3')-VIa*	Plasmid unnamed1-like (strain VB1190 AN: VB1190) *aph(3')-Ia*	Plasmid unnamed (strain T5-67 AN: NZ_CP043569.1 ) - *msr*(*E*), *mph*(*E*), *armA* pMAL-1 like
MLST profiles		ST636	ST492	ST492	ST1	ST636	ST2	ST2

The XDR phenotype of these strains could be correlated with a large number of multidrug efflux pumps genes, identified in all *A. baumannii* genomes (Table 3). Genes associated with resistance to copper (*MmcO*, *CtpA*, copper chaperon, *Bcr*/*CflA*, *cueR*, *pcoB*, *pcoD*, *CusR*, *CopC*, *cutE*, *CorC*), cobalt, zinc, cadmium (*CzcABCD*, *MFP*, *merR*, *CusA*, *CusR*, *CusS*), chromium (*chrA*), arsenic compounds (*ACR3*, *arsC*, *arsH*, *arsR*) and mercury (*merA*, *merC*, *merR*) have been also detected. Also, class 1 integrons, harboring mainly aminoglycoside resistance genes (Table 3) were detected. It is well known that class 1 integrons represent major vehicles enabling the development, accumulation and dissemination of ARGs through horizontal transfer. The association of class 1 integrons with successful *A. baumannii* clones (such as ST2, ST636 and ST492) could explain their high prevalence and potential of transmission among patients. The GR6 plasmid replicon (pACICU2-like) was detected in 2 strains from community-acquired infections (1S, 24S). Another plasmid, untypable by the PCR-based replicon typing, similar to pMAL-1 plasmid (99% similarity) was detected in all but one clinical strains and one ambulatory strain (A07 and 01S). This pMAL-1-like plasmid sequence harbors *bla*_OXA-72_. In the A14 clinical strain, however, the *bla*_OXA-23_ gene was truncated, having a deletion of 658 nucleotides (Table 3).

In 2 strains (10s and 14s), 3 intact prophages (70.8 Kb; 37.5 Kb; 13.5 Kb in strain encoded 10s and 40.3 Kb; 52.6 Kb; 41.7 Kb in strain 14s) were also found, without any relation with the presence of ARGs.

According to the virulence factors database all strains harbor a wide range of virulence genes, encoding for: iron acquisition (e.g. *tonB*), pore-forming toxins (*apkA* and *apkB*), adherence, invasion of epithelial cells (*ompA*), phospholipids degradation (*plc*, *plcC*, *plcD*) and apoptosis induction (Table 3). Two of the strains (14s and A14) contain the *hemO* (hemoxigenase) gene, which was associated with the hypervirulent phenotype of *A. baumannii* LAC 4^19^. The ability to produce various virulence factors of these *A. baumannii* strains could explain their ability to persist and colonize the human host leading to a serious threat for hospitalized patients.

The predicted serological typing scheme for the *A. baumannii* strains analyzed in this study shows some aspects related to their virulence. It is known that the major immunogenic polysaccharide which is produced *A. baumannii* as in important virulence factor is the capsular one (K) and not the somatic antigen O, since the non-capsulated strains don’t cause infections^20^. The Supplementary Fig. 1 shows the genetic variability in the K and O loci, the metrics related to the match confidence, coverage, identity and number of genes, as well the locus type for each of the 7 strains that were sequenced in this study, and their relationships with regards to the subtype.

### Pangenome, phage and genomic islands analysis

#### Genomic Islands

Genomic islands (GI) represent proof of horizontal gene transfer in a population; these DNA segments may integrate into the chromosome of the host and undergo transformation, conjugation or transduction^21^. The GI predictions showed that only 4 of the 7 strains belonging to ST1, ST2 and ST636, harbored genomic islands: 3 with putative type IV secretion system (T4SS) ICEs and one with putative IME.

The strain 01s has a putative T4SS-type ICE 266 kb (~ 38% GC) region, containing 273 ORFs among which some encode for the following components of T4SS (AAA_10, TrbC, TraL, TraE, TraK, TrbI, TraV, TraU, TraN, TraH, TraG), while others for: a Prim-Pol primase-polymerase, Phage integrase, Pfam-B_3022 (which may be an mRNA interferase toxin of the MqsR-MqsA toxin-antitoxin system, and biofilm/motility regulator), TrwB_AAD_bind type IV coupling protein, TrwC relaxase.

The strain 18s has a putative IME region of 31 kb (41%GC) containing 39 ORFs, some of them encoding for TrbI T4SS component, Rep_trans relaxase and *rve* integrase.

The strain 24s has a putative T4SS-type ICE 282 kb (~ 38% GC) region containing 280 ORFs among which some encode for the following T4SS components (TrbH, traP_typeI, traK_typeI, AAA_10, T2SSE, Plasmid_RAQPRD)—traP and traK could be hints for an F-like plasmid in *A. baumannii*. Other ORFs encode for T4CP proteins (such as FtsK_SpoIIIE), DUF1525, relaxases and phage integrases.

The strain A14 has a putative T4SS-type ICE 131 kb (~ 35% GC) region containing 156 ORFs among which some code for the following T4SS components (TraH, traP_typeI, traK, AAA_10, T2SSE, TraG, TraF, TraN, TrbC, TraU, TrbI, TraE, TraL, TrwC, Pfam-B_1474, Plasmid_RAQPRD), rve integrases, T4CP TrwB_AAD_bind, relaxases.

Further, considering the reference genomes used for phylogenetic analysis and the same GI prediction method, the GI were encountered in 19 out of 69 genomes, with sets of genes similar to the selected sequenced samples from the same phylogenetic cluster. While most of the genomes have only one GI region predicted, 3 of the reference genomes had 2 GI regions (Suppl. Table 1).

Most of the genomes with predicted GI belong to the ST1 cluster. Compared with the references from the same subtype, 18s GI exhibits: (i) the lowest number of ORFs, thus fewer known protein types, even though its length is similar with other Putative IME GI from different references; (ii) the highest GC percentage (41.19%) in the genomic island (Suppl. Table 1).

When compared with the reference genomes, the strains 24s and 01s belonging to ST636, and A14 from ST2 have the longest GI, with the highest number of ORFs and similar GC.

Beside this prediction, traces of Tn*AbaR* were also found. Tn*AbaR is* a core composite transposon bound by inverted repeats and 2 copies of direct repeats at its ends, forming an *AbaR* resistance island. Usually, *AbaR*s are inserted in *A. baumannii* genes, leading to the loss of their function—in this case, *comM* (an ATPase-encoding gene known as a hotspot for the integration of *Aba*R). These islands consist mainly of MGEs, such as transposons or integrons, and various genes that confer MDR^22^.

Therefore, of the 7 strains, 4 of them (01s, 14s, 24s and A07) fully cover Tn*AbaR23* (a 50k bp Tn*AbaR*-like island, also containing the partial sequence of *comM* gene—GenBank: JN676148.1) while 2 of them (10s and A14) partially cover it.

Although they fully cover this resistance island, 14s and 01s strains have the lowest overall identity (20% and 25%, respectively) with Tn*AbaR23*. The contig from 01s has 100% identity to the partial sequence of *comM* gene, while 14s has the highest local identity (97.1%) only to *TnpA* IS*15DI* transposase from Module_I. The strains 24s has an overall 47.1% identity with Tn*AbaR23*, though having 92%, 100% and 100% identity to Module_I,_J and _K, respectively, while A07 has an overall 37.2% identity, though having 100% and 83.6% identity to Module_A and _B, respectively.

The other strains that partially cover Tn*AbaR23* also have low identities: 10s has an overall 27.1% identity (with 97.1% identity to *TnpA* gene from Module_I). A14 has an overall 32.8% identity, of which modules A to first half of Module_F have no coverage, the only high similarity region comprising the end of Module_F and the start of Module_G.

On the other hand, the contigs of 18s cover almost entirely Tn*AbaR23*, with 99.97–100% identity to Module_A,_B,_C,_D,_F and _K, 93.4% to Module_G and 36% to Module_I. Almost all of Module_J has no coverage (highlighted in yellow in Suppl. Figure 2): the end part of the *CadR* (transcriptional regulator of MerR family), whole *CadA* (heavy metal transport/detoxification protein), whole *LspA* (prolipoprotein signal peptidase) and almost all of the transposase in this module (protein ID: AFB76410).

The Tn*AbaR*-like island of 18s had most similarities to other Tn*AbaR* islands, mostly on modules A to half of G, and K, given by the backbone transposon Tn*6019* (Suppl. Figure 2). The highest similarity is with Tn*AbaR23* (as it can be seen in the identity distance matrix from Suppl. Figure 2). The region in Module_G (highlighted in cyan in Suppl. Figure 2) is different between 18s/Tn*AbaR23* and the other transposons. The dissimilarity is increased between 18s and the other transposons on the end part of Module_G and most of Module_I (highlighted in purple in Suppl. Figure 2), where the 18s resistance island may acquire structural variation compared to the others.

#### Phages

The most phage-abundant regions were found in 2 strains that belong to the ST492 subtype. In these strains we found the highest number of intact phages, phage species and attachment sites for phages compared to the other strains with other subtypes (Suppl. Table 1).

#### Pangenomes

**Figure 1:**
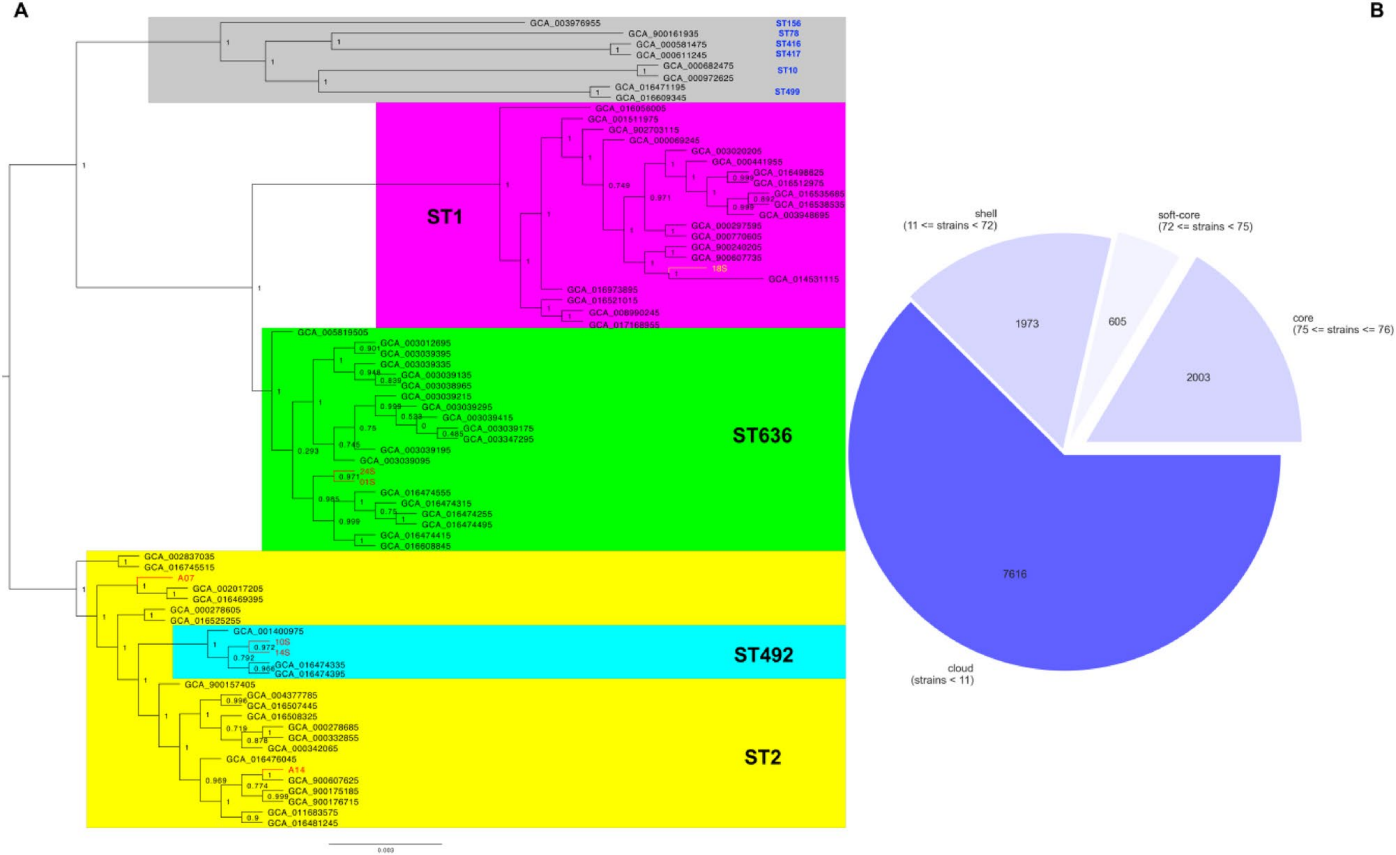
Phylogenetic tree of 76 genomes based on pangenome analysis. (**A**) Phylogenetic clustering; (**B**) The distribution of core and accessory genes in all 76 genomes.

The pangenome analysis shows that the total number of genes found in each of the samples is approximately 3700. Around 43% of these are represented by hypothetical proteins (Fig. 1b). The genomes were subsequently compared one with each other as a matter of unique genes. Then, the lowest numbers taken by subtype belong to ST492 and ST636, while the highest numbers are found in ST2 (6 to 8 times higher than the previous two) and ST1 (almost 3 times higher than in ST2).

The ST492 is of particular interest, considering the subtype is relatively new and not much is known about it in literature. Taking into account that the highest number of phages, but no genomic islands were predicted in two ST492 strains, we took a closer insight into their genome. When compared to the other genomes considered in our phylogenetic analysis, the 2 ST492 strains contain 19 unique genes that were not found in any other genome from the selected ones (including 3 other ST492 reference genomes). Of these 19 genes, 17 are hypothetical and the other 2 are represented by: *dnaB2* (a replicative DNA helicase) and *bfrD1* (a putative TonB-dependent receptor BfrD). These 2 ST492 strains have 62 proteins in common, of which 57 are hypothetical proteins while the other 5 are represented by: *fpvA* (ferripyoverdine receptor), *pucD* (putative xanthine dehydrogenase subunit D), *folE* (GTP cyclohydrolase 1), *bfrD1* and *dnaB2*. When considering all 5 ST492 genomes from the phylogenetic analysis set, then there are 18 proteins unique only to ST492 of which 11 are hypothetical and the other 7 proteins are: *ptk* (tyrosine-protein kinase), *ptp* (low molecular weight protein tyrosine-phosphatase), *glxR2* (2-hydroxy-3-oxopropionate reductase), *wbpA* (UDP-N-acetyl-D-glucosamine 6-dehydrogenase), *pglH* (GalNAc-alpha-(1- > 4)-GalNAc-alpha-(1- > 3)-diNAcBac-PP-undecaprenol alpha-1,4-N-acetyl-D-galactosaminyltransferase), *mshA3* (D-inositol-3-phosphate glycosyltransferase) and *pglA* (N,N'-diacetylbacillosaminyl-diphospho-undecaprenol alpha-1,3-N-acetylgalactosaminyltransferase).

All 76 samples that were used for the phylogenetic analysis have in common approximatively 2000 core genes and present other 8-9000 accessory genes. This suggests that the selected genomes for the phylogenetic and pangenome analysis have some active MGEs, which may contain various types of resistance genes, integrases, transposases—already predicted and mentioned within the body of this manuscript.

#### Phylogenetic analysis

The phylogenetic relationships between the selected 76 genomes show a clear clustering between the subtypes of interest in which the studied strains fit in. It also shows the separate evolution of ST492 from ST2 (as previously mentioned, ST492 and ST636 are single and respectively triple locus variants of ST2)^23^. The other 8 randomly selected subtypes cluster separately (Fig. 1a). Few other studies on ST492 have been mentioned in literature^24, 25^, but the genomes may have not been uploaded in NCBI database^26^, although not much is known about this subtype, therefore only 5 genomes for ST492 have been used for the phylogenetic analysis. The 2 ST636 strains from this study (01S and 24S) group together, separately from other ST636, while the ST492 ones (10S and 14S), even if they are a subbranch of ST2, are in the same cluster with the other two ST2 samples (A07 and A14).

Similar to the phylogenetic relationship and MLST predictions, some of the genes are expressed only in the isolates from the same branch (e.g., *bla*_ADC-74_ predicted to be present only in 01S and 24S isolates (ST636); *bla*_ADC-11_, *bla*_PER-1_ only for A07 and A14 isolates belonging to the ST2 clone; *bla*_ADC-30_ *tet*(*B*), *sul2*—only for 10S and 14S isolates (ST492 clone). Based on the variant results, approximately 1.25% average of the whole *A. baumannii* genome length in each of the 7 strains proved to be variants. At the same time, most of the variants predicted by ARIBA are found in the variant list from *snippy*^27^, in the same or very similar genes.

## Discussions

*A. baumannii* is one of the most successful pathogens responsible for nosocomial infections, occurred especially in patients admitted to intensive care units (ICUs), but also for community-acquired infections, being able to acquire resistance to carbapenems, fluoroquinolones and aminoglycosides^28^. Due to the limited options for the antibiotic treatment of the produced infections, CRAB isolates became a significant health problem worldwide^29^. Although antibiotic resistance may not be considered a traditional virulence factor, in case of *A. baumannii* it is by far the biggest driver of the clinical outcome by precluding the clinician's ability to eliminate the infecting strain. Our experimental data have shown a high prevalence of imipenem resistance (96.96%) among *A. baumannii* strains isolated in 2017–2018 from hospitalized and community-acquired infections in patients from Bucharest. This percentage was close to reported rates in Egypt, Turkey, Spain and Italy and higher than from Saudi Arabia^30–34^.

To date, in Romania, there has been reported a high prevalence of CRAB strains in different parts of the country including Bucharest, the capital city^35^. Even though *bla*_OXA-58_ has been identified in *A. baumannii* (ST1 clone) recovered from patients in our country^36^, the ST2 associated with *bla*_OXA-23_ remains the most common among the CRAB isolates. A pilot study from 3 Romanian hospitals—from Iași and Târgu-Mureș, performed between 2014 and 2015—showed the presence of carbapenemases OXA-23 and OXA-24/72 in *A. baumannii* nosocomial isolates^37^.

In our study, the MLST analysis has revealed that the strains belonged to 6 different clones: ST2 (pulsotypes I, II, III and IX), ST636 (pulsotypes VI and VIII), ST492 (pulsotype VII), ST312 (pulsotype IV), ST642 (pulsotype V) and ST1 (pulsotype VI); ST2 was the most frequently encountered clone (12/33; 36.36%) followed by ST636 (9/33; 27.27%) and ST492 (6/33; 18.18%) (Table 1).

Regarding the distribution of the carbapenemases genes among the identified clones, it has been observed that the ST2 clone encountered in hospital A was associated with the production of *bla*_OXA-23_ gene (10/33; 30.30%) carbapenemase and revealed, in most cases, the presence upstream the carbapenemase gene, of the IS*Aba1* element (9/33; 27.27%). The presence of IS*Aba1* upstream *bla*_OXA-23_ and *bla*_OXA-51_ is required to confer resistance to carbapenems^38^. Although the relationship between IS*Aba1* upstream *bla*_OXA-51_ and carbapenem resistance was confirmed, this might not be enough to confer resistance, as *A. baumannii* isolates susceptible to carbapenems with the association IS*Aba1*/ *bla*_OXA-51_ have already been described^39^. The clone harboring 2 carbapenemases: OXA-23 and OXA-51 was identified in the ambulatory sector (clinical unit B) (Table 1); the ST636 clone was related with the presence of OXA-24 carbapenemase, most of the strains being isolated from hospital C (7/33; 21.21%) and 2 strains from the ambulatory. The other isolates belonging to STs 492, 312, 642 and ST1 were associated with *bla*_OXA-24_ gene (Table 1). The distribution of these clones per hospital unit revealed that ST492 and ST1 were found exclusively in hospital C; ST642 in hospital A, while ST312 was identified in both hospital units (A and C) (Table 1).

Currently, worldwide carbapenem resistant strains are mostly associated with international clone II, with *bla*_OXA-23_ as the main carbapenem resistance mechanism^12^. In Greece, it has been observed that the ST2 was the most common clone circulating in Greek hospital settings^40^. With regards to MDR, several other authors have demonstrated the association of *bla*_OXA-23_, *bla*_OXA-58_, *bla*_OXA-72_ and ST2^24, 41–44^. Furthermore, the international clone ST2 was found to be broadly spread among our country^45^. Very recently Lukovik et al., have reported the same association of CRAB circulating in Serbia: *bla*_OXA-66_/*bla*_OXA-23_/ST2 *bla*_OXA-66_/*bla*_OXA-72_/ST492, and *bla*_OXA-66_/ *bla*_OXA-72_/ST636^25^. These data confirm the multidirectional evolution of the CRAB clones in neighboring countries. A particular case has been noticed in Serbia by Misic et al. in 2018 which have demonstrated the presence of Aci1 carrying *bla*_OXA-72_ belonging to ST1 in a companion animal emphasizing the importance for both animal and public health^46^. Jakovac et al. demonstrated the presence of the international clone II carrying *bla*_OXA-23_ and *bla*_OXA-72_ in nosocomial CRAB recovered from the southwestern part of Bosnia and Herzegovina^47^. A sporadic CRAB clone harboring a unique *bla*_OXA-72_ carrying plasmid have been reported in China from a patient with community-acquired pneumonia^48^.

Similar to our results, the presence of multiple ARGs for β-lactams, aminoglycosides, sulfonamide and tetracyclines was evidenced in clinical MDR *A. baumannii* isolates from Spain and Switzerland^49, 50^.

Some of the identified plasmids (e.g. pACICU-2 like ST2 carrying *bla*_OXA-23_; pMAL-1like ST492 and ST1 carrying *bla*_OXA-72_) were previously described in our country, in *A. baumannii* strains belonging to different clones^35, 51^ and in Serbia (GenBank accession no. KX230793.1), while others (e.g. pACICU-2like ST636 carrying *bla*_OXA-72_ gene; pACICU-2 like and pMAL-1 like ST2 carrying *bla*_OXA-23_) are reported for the first time in community-acquired and intra-hospital *A. baumannii* strains isolated in Bucharest. In our study, due to sequencing limitations, the *bla*_OXA-23_ gene location was not identified. Our data revealed that community-acquired *A. baumannii* harbored pACICU-2 linked to ST636 producing OXA-72; pMAL-1linked to ST492 and ST1 producing OXA-72 and clinical CRAB pACICU-2 like and pMAL-1linked to ST2 OXA-23 producing.

The OC locus prediction shows that the analyzed strains contain 3 locus types: OCL1, OCL2 and OCL4. OCL1, present in 4 out of 7 strains, is known to be the most abundant gene cluster in the major global clone groups GC1 and GC2, of which ST1 and ST2 belong to. In this case the ST1 strain belongs to OCL4 and the ST492 to OCL1 (supporting thus the hypothesis that ST492, although rare, resulted from ST2, as suggested by phylogeny, or vice versa). The major difference is found between the strains belonging to the ST636 subtype, where *wecB* gene, known to be involved in the biosynthesis of sialic acid, occurs between the glycosyltransferases *gtrOC8* and *gtrOC9*^52^.

On the other hand, the diversity is increased in the K loci, the 7 strains containing 5 different gene clusters: KL1, KL3, KL30, KL40 and KL77.

The major differences appear between the capsular export region and the repeat unit of translocase (*wzx*), with the presence or absence of various types of genes: UDP-N-acetylglucosamine-2-epimerase (*mnaA*) and UDP-N-acetylmannosamine dehydrogenase (*mnaB*) in the ST636 strain; UDP-d-GlcpNAcA epimerase (*gne2*) in ST1; d-glucosaminate PTS permease components EIIA, EIIB, EIIC (*dgaA, dgaB, dgaC*) in A14 from ST2; Photosystem I P700 chlorophyll a apoprotein A1 & A2 (*psaA*, *psaB*), Photosystem I iron-sulfur center (*psaC*), Photosystem I reaction center subunit II & IV & III (*psaD*, *psaE*, *psaF*) in A07 from ST2. Actually, A07 strain has the most diverse K locus from the analyzed strains—there is also an acyl/acetyl transferase (e.g. *atr20*) which is inserted in the simple sugar synthesis region. More than that, the insertion of *atr20* may suggest a novel K locus even though it matches the K77 reference, according to the *Kaptive* documentation. Similarly may happen to the K loci in ST492 strains, since there is no coverage in the respective region for *wzy*, *gtr63* and *itrA2*; this finding, together with the low matching confidence and missing genes between *gna* and *wzx* compared with the other samples, is suggesting a possible novel variant. We also revealed that the p-ACICU-2like and pMAL-1like carrying *bla*_OXA-23_, p-ACICU-2like carrying *bla*_OXA-72_ and pMAL-1like carrying *bla*_OXA-72_ presented virulence-related genes involved in iron acquisition (such as the *TonB*-dependent receptor), adherence (*ompA*), biofilm formation (*pgaABCD* locus), invasion (septicolysin) and haemolytic activity against human erythrocytes, aiding in iron acquisition) (e.g. *plc*, *plcC* and *plcD*)^53^. TonB-dependent transporters are outer membrane proteins (OMPs) that bind and transport siderophores in addition to vitamin B12, nickel complexes, and carbohydrates and may be involved in the survival of bacteria in the lungs central nervous system and blood^54^. Septicolysin, on the other hand, is a pore-forming toxin with a cytolytic activity that mediates invasion of tissues or cells^55^.

## Materials and methods

### Bacterial isolates

Thirty-three carbapenemase-producing *A. baumannii* were isolated from hospitalized, ICU patients (n = 30) and ambulatory patients (3) during Sept. 2017–Feb. 2018 and identified by commercial systems (VITEK2, Microscan and Maldi TOF) (Table 1).

### Susceptibility testing

The susceptibility of *A. baumannii* strains to meropenem (MEM—10 µg), imipenem (IMP—10 µg), ertapenem (ETP—10 µg), cephalotin (CEF—30 µg) ceftriaxone (CTX—30 µg), cefuroxime (CXM—30 µg), cefoxitin (FOX—30 µg), ceftazidime (CAZ—30 µg), aztreonam (ATM—30 µg), cefepime (FEP—30 µg), amoxicillin-clavulanic acid (AMC—10 µg), piperacillin-tazobactam (PIP-TZP—10 µg), ciprofloxacin (CIP—5 µg), levofloxacin (LEV—5 µg) gentamicin (GEN—5 µg), amikacin (AMK—5 µg), nitrofurantoin (NIT—300 µg), trimethoprim-sulfamethoxazole (SXT—23.75 µg), tetracycline (TET—30 µg), tigecycline (TIG) and colistin (COL) (Table 1) was tested by disk diffusion and microdilution methods (for MEM, COL and TIG assessed by broth microdilution method with Mueller–Hinton broth), according to Clinical and Laboratory Standards Institute (CLSI), 2018 and 2019 guidelines^56, 57^. Strains displaying MICs > 8 mg/L for MEM; MICs > 4 mg/L for COL and MICs > 2 mg/L for TIG according EUCAST breakpoints for *Enterobacterales* were considered resistant^58^. *Pseudomonas aeruginosa* ATCC 27,853 was used as a quality control strain. *A. baumannii* were classified as follows: multidrug-resistant (MDR) [resistant to at least one agent in 3 or more antimicrobial categories], extensively drug-resistant (XDR) [resistant to at least one agent in all, but 2 or fewer antimicrobial categories] and pandrug-resistant (PDR) [resistant to all agents in all antimicrobial categories tested^59^.

### PCR for carbapenemase genes

Genes encoding common class D carbapenemases (*bla*_OXA-51-like_, *bla*_OXA-58-like_, *bla*_OXA-23-like_, *bla*_OXA-24-like_, *bla*_OXA-143-like_ and *bla*_OXA-235-like_) and class B metallo-β-lactamases (*bla*_IMP_, *bla*_VIM_, *bla*_NDM_, *bla*_OXA-48_) were detected by PCR^60, 61^, followed by sequencing of selected amplicons. The presence of IS*Aba1* upstream of *bla*_OXA-23-like_, *bla*_OXA-24-like_ was tested by PCR and sequencing using custom-designed primers (OXA-24A: CTCTAAGCCCCAAAATTTCC; OXA-24B: CGCATAAGGCGTATTATGTTA; ISAbaF1: CCTCAGTTTAATGCCAATGCT). The increased expression of *bla*_OXA-51_ genes was analyzed by PCR for IS*Aba1* upstream of the gene^35^. Sequence analysis was performed using Chromas Lite 2.1 software and compared with sequences deposited in the GenBank database^26^.

### Pulsed-field gel electrophoresis (PFGE)

The clonality of *bla*_OXA-23_ and *bla*_OXA-24_ carrying *A. baumannii* was determined with PFGE using the ApaI enzyme and CHEF-DRII apparatus (Bio-Rad). Pulsotypes were defined as isolates with PFGE band patterns of 85% similarity or above^62^. The isolates were classified according to the criteria described^63^.

### Mating experiments

Transferability of *bla*_OXA-23_ by conjugation was tested in solid mating using a rifampicin (RIF)—resistant *Acinetobacter baylyi* ADP1 as a recipient. Briefly, equal amounts (100 µL) of overnight cultures of the donor (A14; A17; 8A) and recipient strains were mixed and incubated in Brain heart infusion agar plates, cells were resuspended in saline solution and selected in plates containing RIF (300 mg/L) and MEM (0.5 mg/L)^64^. Characterization of the transconjugants was conducted by PCR to confirm the conjugative transfer.

### Multilocus sequence typing (MLST)

MLST was used to establish the clonal relationships in *A. baumannii* representative strain from each pulsed-field type, using 7 housekeeping loci (elongation factor EF-G [*fusA*], citrate synthase [*gltA*], CTP synthase [*pyrG*], homologous recombination A factor [*recA*], 60-KDa chaperonin [*cpn 60*], 50S ribosomal protein L2 [*rplB*] and RNA polymerase subunit B [*rpoB*] according to the Institute Pasteur scheme^65^.

### Whole-genome sequencing and bioinformatic protocols

The genomes of 7 *A. baumannii* strains isolated from different infections were sequenced using HiSeq and MiSeq X Ten, Illumina. The paired-end raw reads have been submitted to the GenBank under accession numbers: SRX4094320, SRX4094321, SRX4094322, SRX4094323, SAMN14525745, SAMN14525746, SAMN14525747 and were subsequently assembled using *shovill*^66^ with SPADES 3.12.0^67^ already implemented, while the quality of assembling was checked with QUAST^68^. The samples underwent a reference mapping step in Geneious Prime 2020.1.1^69^, with trimming in a 5 iteration step and taking into account only the reads with a quality factor higher than Q30 resulting in coverage of approximatively 35x-to-38x on the whole genome. Annotations were performed with RAST^70^ and Prokka^71^ while the prediction of resistance and virulence profiles was performed by using the following programs and databases: Abricate (compared against VFDB database for virulence factor prediction), ARIBA (downloading and preparing at first the reference data from NCBI database), ResFinder (compared against implemented NCBI database), PlasmidFinder (compared against implemented plasmidfinder database), PathogenFinder, CARD, PubMLST, ISfinder, PATRIC^72–83^. The resistance genes and virulence factors were selected based on the highest identity and coverage. Plasmid replicons were detected using AB-PBRT^84^ by an in silico PCR approach sequences^85^. The untypable plasmids were compared with the NCBI nucleotide database, while the plasmids integrated within the bacterial chromosome were detected using the BLAST tool^86^ using a command-line interface. All the other predictions were run by using default parameters.

Integron analysis was performed using command-line blastn^86^, with 5’CS and 3’CS sequences (Supplementary file 1) as queries against assembled genomes. Integron sequences annotations were performed manually.

Additional plasmids analyses were performed using the PLSDB database Search tool^87^, using the Mash strategy, with search parameter set as default (max. p-value at 0.1 and min. identity 0.99).

The phylogenetic analysis was performed using the following protocol: (i) taking into account the subtyping information from MLST prediction for the 7 strains discussed in our study, the assembly contigs from 69 reference sequences were selected from the NCBI database^26^. After repeating the MLST prediction on the majority of *A. baumannii* genomes from NCBI (that were uploaded as contigs) the selection of the 69 sequences used for phylogeny was done randomly. Approximatively 20 genomes were selected from each subtype corresponding to the subtypes found in our samples (except ST492—only 3 out of ~ 6000 genomes were found) while other 8 genomes belong to different subtypes. (ii) Whole genome annotation with Prokka (using genetic code 11 and Genbank/ENA/DDJB compliance options) was performed for each of the 76 genomes selected for phylogeny. (iii) the output from Prokka was used for pangenome analysis using Roary^88^. (iv) the phylogenetic tree was generated (on the alignment of core genome gene sequences in Roary with MAFFT^89^, using FastTree^90^ with Jukes-Cantor model, Maximum-likelihood and minimum-evolution NNIs and SPRs algorithms, implemented in Geneious Prime 2020.1.1. The phylogenetic tree image was generated using FigTree software (http://tree.bio.ed.ac.uk/software/figtree/). The Genomic Islands predictions were performed with ICEfinder online tool^91^, while the phage predictions were done with PHASTER online tool^92^. K and OC locus predictions were performed using the Kaptive software^20^. Tn*Aba*R sequences have been aligned with MAFFT, implemented in Geneious Prime.

## Conclusions

Our results highlight the presence of an impressive armamentarium of ARGs and of mobile elements required to form their mobilization and transmission, such as the plasmids, transposons and ISs in CRAB strains isolated from Romanian patients. These strains belong to widespread clones reported in intrahospital infections or community-acquired patients. The most frequently encountered clones identified in *A. baumannii* clinical strains were ST2, ST636 and ST492, while the most frequent carbapenemase genes were *bla*_OXA-23_ or *bla*_OXA-24_. Among resistance determinants, several virulence genes, as well as factors that contribute to the persistence of these bacteria in the hospital environment, have been detected. All sequenced *A. baumannii* isolates have the genetic equipment conferring them the ability to grow in iron-depleted media and to survive in the presence of desiccation, antimicrobials and toxic compounds. Our data will facilitate the understanding of resistance, virulence and transmission features of XDR AB isolates from Romanian patients and might contribute to the implementation of appropriate infection control measures for limiting the spread and decreasing the infection rate and mortality.

## Supplementary Information

Supplementary Information 1.

Supplementary Information 2.

Supplementary Information 3.

Supplementary Information 4.

Supplementary Information 5.

## Data Availability

*A. baumannii* strains are available from the authors.
