# Supplementary material for: Subtypes, resistance and virulence platforms in extended-drug resistant Acinetobacter baumannii Romanian isolates
Source: Sci Rep. 2021 Jun 24;11:13288. doi: 10.1038/s41598-021-92590-5 (PMC8225882; doi:10.1038/s41598-021-92590-5)
Supplement: Supplementary file 1 — Supplementary Information 1. [file 41598_2021_92590_MOESM1_ESM.docx]

Supplementary file. Sequences used as queries for 5’-CS and 3’-CS regions for integrons identification

>5CS
tcaggcaacgacgggctgctgccggccatcagcggacgcagggaggactttccgcaaccggccgttcgatgcggcaccgatggccttcgcgcaggggtagtgaatccgccaggattgacttgcgctgccctacctctcactagtgaggggcggcagcgcatcaagcggtgagcgcactccggcaccgccaactttcagcacatgcgtgtaaatcatcgtcgtagagacgtcggaatggccgagcagatcctgcacggttcgaatgtcgtaaccgctgcggagcaaggccgtcgcgaacgagtggcggagggtgtgcggtgtggcgggcttcgtgatgcctgcttgttctacggcacgtttgaaggcgcgctgaaaggtctggtcatacatgtgatggcgacgcacgacaccgctccgtggatcggtcgaatgcgtgtgctgcgcaaaaacccagaaccacggccaggaatgcccggcgcgcggatacttccgctcaagggcgtcgggaagcgcaacgccgctgcggccctcggcctggtccttcagccaccatgcccgtgcacgcgacagctgctcgcgcaggctgggtgccaagctctcgggtaacatcaaggcccgatccttggagcccttgccctcccgcacgatgatcgtgccgtgatcgaaatccagatccttgacccgcagttgcaaaccctcactgatccgcatgcccgttccatacagaagctgggcgaacaaacgatgctcgccttccagaaaaccgaggatgcgaaccacttcatccggggtcagcaccaccggcaagcgccgcgacggccgaggtcttccgatctcctgaagccagggcagatccgtgcacagcaccttgccgtagaagaacagcaaggccgccaatgcctgacgatgcgtggagaccgaaaccttgcgctcgttcgccagccaggacagaaatgcctcgacttcgctgctgcccaaggttgccgggtgacgcacaccgtggaaacggatgaaggcacgaacccagttgacataagcctgttcggttcgtaaactgtaatgcaagtagcgtatgcgctcacgcaactggtccagaaccttgaccgaacgcagcggtggtaacggcgcagtggcggttttcatggcttgttatgactgtttttttgtacagtctatgcctcgggcatccaagcagcaagcgcgttacgccgtgggtcgatgtttgatgttatggagcagcaacgatgttacgcagcagggcagtcgccctaaaacaaag

>3CS
ttagatgcactaagcacataattgctcacagccaaactatcaggtcaagtctgcttttattatttttaagcgtgcataataagccctacacaaattgggagatatatcatgaaaggctggctttttcttgttatcgcaatagttggcgaagtaatcgcaacatccgcattaaaatctagcgagggctttactaagcttgccccttccgccgttgtcataatcggttatggcatcgcattttattttctttctctggttctgaaatccatccctgtcggtgttgcttatgcagtctggtcgggactcggcgtcgtcataattacagccattgcctggttgcttcatgggcaaaagcttgatgcgtggggctttgtaggtatggggctcataattgctgcctttttgctcgcccgatccccatcgtggaagtcgctgcggaggccgacgccatggtgacggtgttcggcattctgaatctcaccgaggactccttcttcgatgagagccggcggctagaccccgccggcgctgtcaccgcggcgatcgaaatgctgcgagtcggatcagacgtcgtggatgtcggaccggccgccagccatccggacgcgaggcctgtatcgccggccgatgagatcagacgtattgcgccgctcttagacgccctgtccgatcagatgcaccgtgtttcaatcgacagcttccaaccggaaacccagcgctatgcgctcaagcgcggcgtgggctacctgaacgatatccaaggatttcctgaccctgcgctctatcccgatattgctgaggcggactgcaggctggtggttatgcactcagcgcagcgggatggcatcgccacccgcaccggtcaccttcgacccgaagacgcgctcgacgagattgtgcggttcttcgaggcgcgggtttccgccttgcgacggagcggggtcgctgccgaccggctcatcctcgatccggggatgggatttttcttgagccccgcaccggaaacatcgctgcacgtgctgtcgaaccttcaaaagctgaagtcggcgttggggcttccgctattggtctcggtgtcgcggaaatccttcttgggcgccaccgttggccttcctgtaaaggatctgggtccagcgagccttgcggcggaacttcacgcgatcggcaatggcgctgactacgtccgcacccacgcgcctggagatctgcgaagcgcaatcaccttctcggaaaccctcgcgaaatttcgcagtcgcgacgccagagaccgagggttagatcatgcctagcattcaccttccggccgcccgctagcggaccctggtcaggttccgcgaaggtgggcgcagacatgctgggctcgtcaggatcaaactgcactatgaggcggcggttcataccgcgccaggggagcgaatggacagcgaggagcctccgaacgttcgggtcgcctgctcgggtgatatcgacgaggttgtgcggctgatgcacgacgctgcggcgtggatgtccgccaagggaacgcccgcctgggacgtcgcgcggatcgaccggacattcgcggagaccttcgtcctgagatccgagctcctagtcgcgagttgcagcgacggcatcgtcggctgttgcaccttgtcggccgaggatcccgagttctggcccgacgccctcaagggggaggccgcatatctgcacaagctcgcggtgcgacggacacatgcgggccggggtgtcagctccgcgctgatcgaggcttgccgccatgccgcgcgaacgcaggggtgcgccaagctgcggctcgactgccacccgaacctgcgtggcctatacgagcggctcggattcacccacgtcgacactttcaatcccggctgggatccaaccttcatcgcagaacgcctagaactcgaaatctaacgtccgttcgggcatcgaggtccatgtcggggtgggacgggcccgtggcttcaagatcacttgcagtccgaccgcgatgtcttggttgcgcgagaggttgtcgaca
